# Cushing’s Syndrome and a Dead Fetus in Pregnancy: A Clinical Case and Behavior Report

**DOI:** 10.7759/cureus.56769

**Published:** 2024-03-23

**Authors:** Zhivko Zhekov, Svetlana Y Radeva, Yanko G Yankov

**Affiliations:** 1 Department of Gynecology, Specialized Hospital of Obstetrics and Gynecology for Active Treatment, Varna, BGR; 2 Department of Obstetrics and Gynecology, Medical University "Prof. Dr. Paraskev Stoyanov", Varna, BGR; 3 Faculty of Public Health, Medical University "Prof. Dr. Paraskev Stoyanov", Varna, BGR; 4 Clinic of Maxillofacial Surgery, University Hospital "St. Marina", Varna, BGR; 5 Department of General and Operative Surgery, Medical University "Prof. Dr. Paraskev Stoyanov", Varna, BGR

**Keywords:** adrenocortical adenoma, sectio parva, foetus mortus, pregnancy, cushing syndrome

## Abstract

Cushing’s syndrome is a rare disease that has a different primary etiology, most often due to chronic hypercortisolism. In addition to the defined causes, in contrast to the general population, the observed etiology in pregnant women is a benign adrenocortical adenoma, less often bilateral hyperplasia of the adrenal glands of hypothalamic-pituitary origin or Cushing’s disease, and malignant adrenal root adenoma. In this study, we present the case of a 41-year-old pregnant woman experiencing her first pregnancy. Her general diseases from anamnesis were chronic obstructive pyelonephritis, acute rhythm disturbance, somnolence, pituitary adenoidectomy, and adrenalectomy of both adrenal glands. The patient was obese, with a BMI of 31.25 kg/m^2^. She sought medical help due to fatigue, anuria, vomiting, a fever of up to 38.9°C, and hypertension. In the 18th gestational week, fetal death was diagnosed. The fever persisted for several days, and the patient had a malaise and became intoxicated with evidence of sepsis. She was hospitalized at two medical facilities for clarification. In the Department of Gynecology at the Specialized Hospital for Obstetrics and Gynecology in Varna, Bulgaria, a cesarean section was performed. The patient’s condition remained stable after surgery. She was referred to a central intensive care unit for follow-up.

## Introduction

Corticosteroid excess, which causes a range of different clinical conditions, was first described by Harvey Cushing as Cushing’s syndrome [1]. Cushing’s syndrome is a rare process during pregnancy, given the high rate of infertility it causes, so there are few reported cases of this disease during pregnancy and their follow-up [2-5]. During pregnancy, there is a change in steroid metabolism, leading to physiological and adaptive hypercortisolism [6]. The total concentration of cortisol increases due to the greater synthesis of its transport protein due to the effect of estrogen stimulation. The circadian rhythm of cortisol is maintained. Adrenocorticotropic hormone levels rise progressively throughout pregnancy, despite elevated cortisol levels, due to ectopic secretion (of placental origin) that is not controlled by cortisol [7]. Female fertility is influenced by various factors, both biological and environmental [8]. Age is an important factor, with fertility declining significantly after the age of 35. Ovulatory disorders such as polycystic ovary syndrome, hormonal imbalances, uterine and cervical abnormalities, fallopian tube damage, endometriosis, and primary ovarian failure can affect fertility [9-11]. Correct diagnosis and subsequent treatment in Cushing’s syndrome and high-risk pregnancy are key due to greater maternal-fetal morbidity and mortality, as in the present case we are describing [12,13].

## Case presentation

We present the case of a 41-year-old female patient with visible signs of aging corresponding to her biological age. She had abdominal obesity, with a BMI of 31.25 kg/m^2^. She was of good social status and of working age, and she had undergone many years of unsuccessful attempts to conceive. No infertility studies, stimulations, or inseminations had been conducted, and no information regarding a partner was available.

The patient was admitted to the Department of Gynecology at the Specialized Hospital of Obstetrics and Gynecology for Active Treatment in Varna, Bulgaria, presenting with somnolence, lumbar pain, anuria, vomiting, and fever reaching 38.9°C for a day. Hydronephrosis was diagnosed, and a specialist obstetrician-gynecologist noted the absence of fetal cardiac activity. Her past regular menstruation was on November 22, 2022, with a confirmed estimated due date of August 29, 2023. Despite 72 hours of observation, the cause of the fever and deterioration in her condition remained undiagnosed. Consequently, the patient was transferred to the University Multispecialty Hospital for Active Treatment “St. Marina” in Varna, Bulgaria, for further evaluation and management. She was admitted to the Clinic of Nephrology.

An abdominal ultrasound examination revealed no evidence of ascites or aerocoli. The liver displayed a normal echogenic structure with non-dilated vessels, intrahepatic bile ducts, gallbladder, and pancreas devoid of calculi or abnormalities. The spleen was of normal size, and the bladder was empty. An amniotic sac without cardiac activity was observed.

Subsequent imaging examinations (roentgenographic) of the lungs and heart in the supine position showed symmetrical findings with an anterior-posterior projection. Diaphragmatic domes were identifiable by their normal positions, convexity, and outlines. Pulmonary blood flow appeared to have increased. Hilus and suprahillus hypervolemias with congestive changes were noted. No inflammatory infiltrates, focal shadows, or nodular shadows were observed in the visible pulmonary tracts. The superior mediastinum appeared unexpanded, and the heart shadow was centrally located. The subdiaphragmatic space appeared normal. Long-standing fractures of the sixth right dorso-axillary rib were visible without evidence of focal lesions.

As the patient’s nephrological symptoms improved, she was transferred to the Clinic of Anesthesiology and Intensive Care. Upon admission, she was conscious but displayed tachypnea, with a respiratory rate of up to 30 breaths per minute. She was placed on an oxygen mask. Her heart rate was 125 bpm, and her blood pressure was 80/40 mmHg. She appeared to be in a visibly compromised general condition and exhibited somnolence. Following an extensive consultation involving various specialists, a unanimous decision was made to proceed with the extraction of the dead fetus to aid in the process of controlling the underlying pathology.

**Figure 1:**
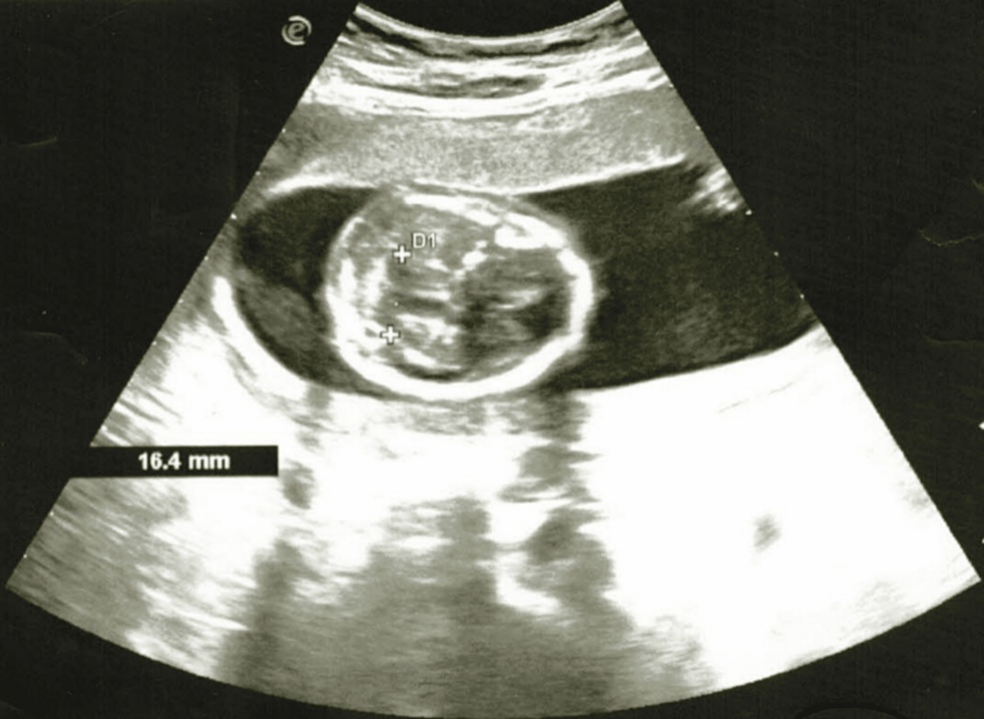
Ultrasound picture of the VPD: 40.0 mm (reference limits: 32-47 mm) VPD, verified parietal diameter

**Figure 2:**
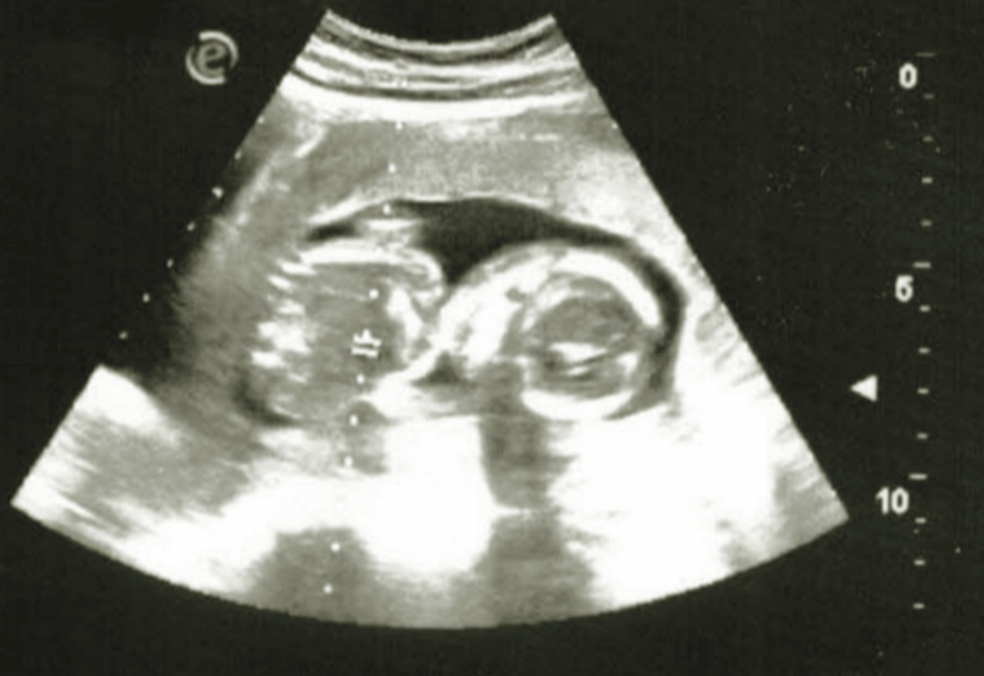
Ultrasound picture of the AC: 129.9 mm (reference limits: 104-144 mm) AC, abdominal circumference

**Figure 3:**
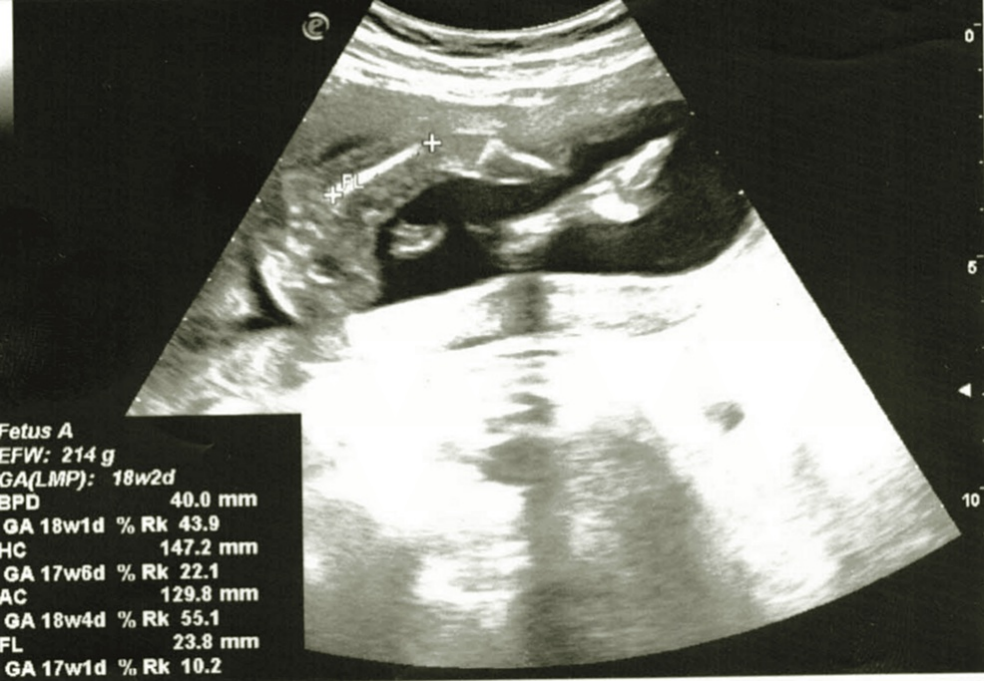
Ultrasound picture of the FL: 23.8 mm (reference limits: 18-32 mm) FL, femur length

On the fifth day, due to the worsening of her general condition, the patient was transferred for obstetric care to the Department of Gynecology at the Specialized Hospital of Obstetrics and Gynecology for Active Treatment in Varna, Bulgaria. During an objective examination at the specialized hospital for obstetrics and gynecology, it was determined that the gestational age corresponded to the period of amenorrhea. She was admitted in a visibly medium-severe compromised condition. Objectively, she exhibited dry skin, pallor with reduced turgor, malaise, a moon-like face, and swelling of the limbs. She was disoriented, with a blood pressure of 180/110 mmHg, a heart rate of 120 beats/min, and a body temperature of 38°C. Ultrasound examination of the fetus revealed the following parameters: verified parietal diameter: 40.0 mm (reference limits: 32-47 mm) (Figure 1); head circumference: 147.2 mm (reference limits: 131-161 mm); abdominal circumference: 129.9 mm (reference limits: 104-144 mm) (Figure 2); femur length: 23.8 mm (reference limits: 18-32 mm) (Figure 3); and placentae: by fundus and anterior wall, fetal heart rate: missing, liquor amnii: moderately reduced, probably weight: 214 g (reference limits: 220-230 g).

From the paraclinical examination, signs of infection and sepsis can be determined (Table 1).

**Table 1:** Blood test analysis report H, lighter than reference limits; L, lower than reference limits INR, international normalized ratio

Parameters	Values		Reference limits
Leukocytes	23.13 × 10^9^ g/l	H	3.5-10 × 10^9^ g/l
Lymphocytes	2.30%	H	0.2-0.4%
Hematocrit	0.295 l/l	L	0.36-0.50 l/l
Hemoglobin	105 g/l	L	120-160 g/l
Erythrocytes	3.57 × 10^12^/l		3.5-5.5 × 10^12^/l
Thrombocytes	294 × 10^9^/l		140-440 × 10^9^/l
Plateletcrit	0.25%		0.11-0.28%
Glucose	7.35 mmol/l	H	3.8-5.8 mmol/l
Creatinine	244.3 mmol/l	H	58-96 mmol/l
Blood urea nitrogen	19.7 mmol/l	H	2.6-7.2 mmol/l
Serum glutamic-oxaloacetic transaminase	13 U/l		0-37 U/l
Serum glutamic pyruvic transaminase	17 U/l		0-41 U/l
Gamma-glutamyl transferase	19 U/l		0-32 U/l
Total bilirubin	34 umol/l	H	5-21 umol/l
C-reactive protein	57.9 mg/l	H	0-5 mg/l
Na^+^	140 mmol/l		135-145 mmol/l
K^+^	3.5 mmol/l		3.5-5.0 mmol/l
Fibrinogen	1.5 g/l	L	2-4 g/l
INR	1.66		0.7-1.3
Bleeding time	150 seconds		120-300 seconds
Clotting time	300 seconds		300-600 seconds

From the examination of the urine, disturbed indicators are observed due to the impaired excretory function (Table 2).

**Table 2:** Urine test analysis report

Urine	Levels	Reference limits
Specific weight	1.02 mIU/mL	1.005-1.030 mIU/mL
рН	5.5	4.8-7.4
Protein	Negative (-)	Negative (-)
Blood test line	Са (+) and 10 erythrocytes/ul	Negative (-)
Leukocytes test line	Са (2+) and 125 leukocytes/ul	Negative (-)
Sediment	5-6 erythrocytes and 15-16 leukocytes	Negative (-)

The patient’s clinical behavior was discussed by an extended council of doctors with different specialties, who recommended performing a cesarean section to evacuate the dead fetus.

The administered therapy included Sterofundin (1 × 1,000 mL IV), serum glucose 5% (1 × 1,000 mL IV), Ringer (× 1,000 mL IV), Famotidine (2 × 0.02 g IM), antibacterial therapy with Ceftriaxone (2 × 2 g IV), and Metronidazole (2 × 0.5 g IV) for three days; Norepinephrine (2 × 0.004 g IV on the first day and 1 × 0.004 g IV on the second day), Methylprednisolone (1 × 0.04 g IV), Furosemide (2 × 0.02 g IV on the first day and 1 × 0.02 g IV on the second day), Tramadol (2 × 0.1 g IM), Metamizole sodium (3 × 1 g IM) for two days; and potassium chloride 150 mg/mL (4 × 10 E IV on the first day), Oxytocin 5 IU/mL (2 × 5 E on the second day), human fibrinogen (1 × 1 g IV), Chloropyramine (1 × 0.02 g IV) and Promethazine (1 × 0.025 g IV) for one day.

The operative intervention was performed under general endotracheal anesthesia. After disinfection, the abdomen was opened by Pfannenstiel. A dead male fetus corresponding to the 18th-19th gestational week with a weight of approximately 170 grams was extracted, with initial signs of maceration and a height of 20 cm. An instrumental revision was performed, and the uterus was closed in two layers, layer by layer. The restoration of the abdominal wall was carried out in reverse order. An intra-abdominal drain was applied. During the surgical intervention, 900 mL of plasma substitute medication (plasma 650 mL and leukocyte-free blood 250 mL) was administered. The abdominal cavity was cleared of blood clots, hemostasis was achieved, and closure was achieved layer by layer in reverse order. About 150 mL of blood loss was reported. An aesthetic suture with polyfilament sutures was placed on the skin. A total of 600 mL of clear urine leaked from the catheter from the time of the placement until the woman was taken to intensive care for five days. The values of blood indicators and urine improved, and she was dismissed.

Control examinations were carried out on the 20th, 30th, and 90th days; the patient was in good general condition and afebrile. The uterus corresponded to the period of the puerperium. The operative wound was calm and healed primarily, and the edges were smooth. The swelling had decreased. Her blood pressure values were normal, and the uterine involution was complete. The operative wound was calm.

**Figure 4:**
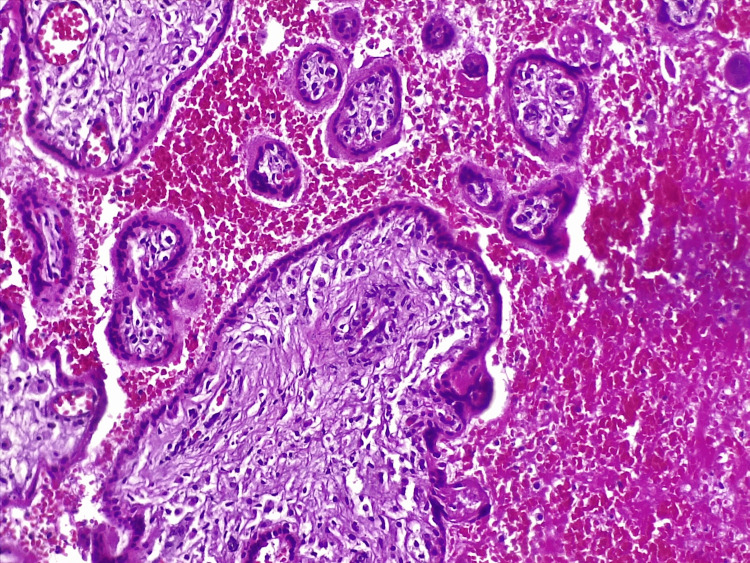
Histopathological examination of placenta material (H&E x40)

**Figure 5:**
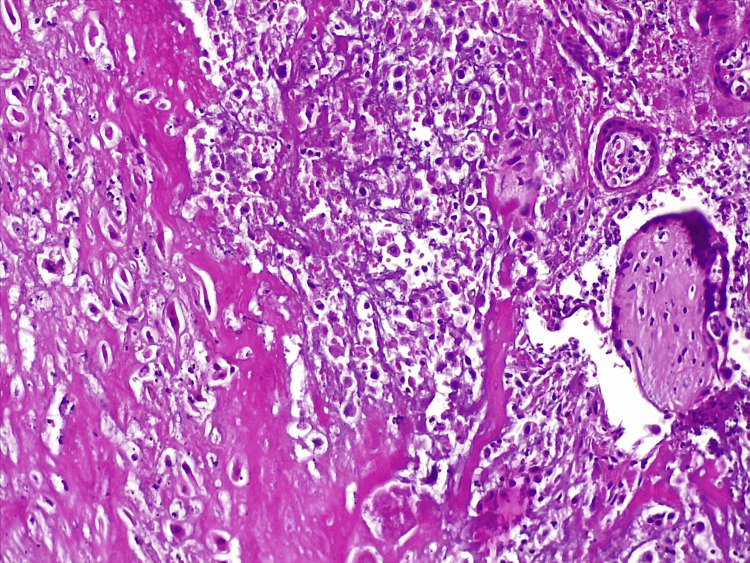
Histopathological examination of placenta material (H&E x40)

**Figure 6:**
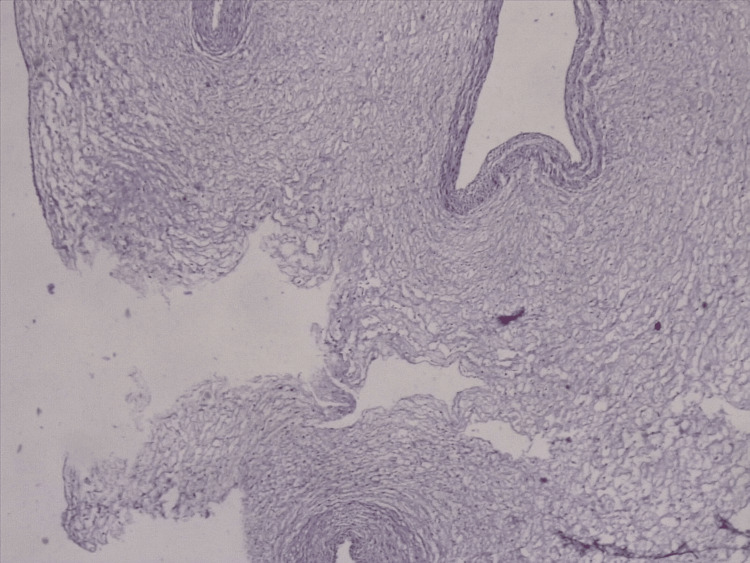
Histopathological examination of umbilical cord material (H&E x4)

A histopathological evaluation of a native preparation of part of the placenta (Figure 4, Figure 5) and umbilical cord (Figure 6) was performed. Hemorrhages with massive areas of necrosis were observed. There were regressive changes in the chorioepithelioma in the form of impaired differentiation of cellular and syncytial trophoblasts, desquamation, thinning, and the complete disappearance of the epithelial cover. Accumulation of fibrin in the intervillous space, petrification of fibrin, and areas of necrosis in the decidual tissue and villi were found. Fatty degeneration, vacuolization, and pyknosis of cells were observed in the decidual tissue. White infarcts were visualized along the edge of the placenta - dense areas of white or yellowish-white color up to 0.3-0.5 cm in diameter. The established pathological processes were characteristic of the morphological picture in a non-developing pregnancy. The established changes in the umbilical cord referred to growth and outflow of the stroma, with atrophy of blood vessels.

In conclusion, a timely diagnostic assessment of the patient’s condition was performed. Therapeutic measures were taken to control the condition, including the evacuation of the dead fetus and subsequent treatment of the main and general diseases of the patient.

## Discussion

In their studies, Pourali et al. and Lindsay et al. present clinical cases of pregnant patients who have the typical picture of obesity (all cases reviewed were ΒMI over 30) and high blood pressure [6,9]. It is not uncommon for patients to develop preeclampsia. In some of the cases presented, HELLР syndrome is developing early detection of abnormalities in patients with characteristic features such as swelling, sharp weight gain, fatigue, and inflammatory processes on the skin, which directs obstetricians to look for endocrine causes of aggravation of pregnancy. These patients experienced changes in their menstrual cycle, difficulties getting pregnant, and severe pregnancy.

In other studies, common symptoms include personality changes, tiredness, and skin manifestations (inflammations, stretch marks, and swelling) [4,6,10]. Up to 60% may have impaired glucose tolerance. In these pregnancies, complications between the mother and fetus are often observed. The manifest manifestations of the infection mask many of the symptoms, and the presence of high glucose levels delays treatment over time.

Early and closely monitored pregnancies of women with Cushing's syndrome, regardless of when it is diagnosed, help to strictly monitor fetal development and bring the pregnancy to a later date with the birth of a live child. In the case presented by us, the pregnant woman is not adequately monitored due to her unwillingness, despite the absence of a previous pregnancy. Because of this, she developed a clinical picture of sepsis, and at 18 weeks of pregnancy, doctors and specialists found a dead fetus.

Age is an important factor, with fertility declining significantly after the age of 35. Ovulatory disorders such as polycystic ovary syndrome, hormonal imbalances, uterine and cervical abnormalities, fallopian tube damage, endometriosis, and primary ovarian failure can affect fertility [9-11].

Blanco et al. reported a case of pregnancy during Cushing’s syndrome [12]. They successfully controlled hypercortisolism with metyrapone, which began at eight weeks of gestation. At the 16th week of pregnancy, they successfully perform a laparoscopic adrenalectomy. Histopathological examination of the gland revealed a benign adrenocortical adenoma. Adrenal insufficiency was treated with daily glucocorticoids. Finally, there was spontaneous vaginal delivery at the 30th week of gestation.

Correct diagnosis and subsequent treatment in Cushing’s syndrome and high-risk pregnancy are key due to greater maternal-fetal morbidity and mortality [12,13]. Perinatal complications include fetal growth restriction, premature birth, stillbirth, and neonatal death. Treatment plans depend on the gestational age, the experience of the clinician, the structure, and the general condition of the patient.

Examining this case highlights the possibilities of the treatment structures to implement the correct medical behavior and the effectiveness of the treatment strategy undertaken to solve emerging problems and complications. The implementation of a correct approach must be based on scientific results to guide clinicians in choosing the most appropriate technologies based on the unique needs of each patient. The incensement of the age of a woman and performing reproductive functions is associated with a number of risks for the embryo and its proper development, which can cause problems with implantation, spontaneous abortions, and the birth of a child with various syndromic conditions.

## Conclusions

Cushing’s syndrome should be considered in pregnant hypertensive patients with notable signs of hypercortisolism. The best results are achieved through the formation of multidisciplinary collaboration teams between obstetricians, endocrinologists, and surgeons. Timely diagnosis in primary care requires high cognitive capabilities of the medical staff in terms of early diagnosis, proper assessment of the patient’s needs, and the consequences of performing a certain treatment.

This report describes the successful collaboration between different structures and the proper assessment of the condition, minimizing the risks to the patient and reducing the burden of hospital costs. The findings suggest that a personalized approach requires good knowledge of healthcare professionals and adequate behavior to address specific challenges in medicine, highlighting the importance of individualized approaches in obstetric care.
